# Analysis of differential gene expression in colorectal cancer and stroma using fluorescence-activated cell sorting purification

**DOI:** 10.1038/sj.bjc.6604931

**Published:** 2009-04-28

**Authors:** M J Smith, A C Culhane, M Donovan, J C Coffey, B D Barry, M A Kelly, D G Higgins, J H Wang, W O Kirwan, T G Cotter, H P Redmond

**Affiliations:** 1 Department of Academic Surgery, University College Cork, Cork, Ireland; 2 Department of Biochemistry, University College Cork, Cork, Ireland; 3 Department of Biostatistics and Computational Biology, Dana-Farber Cancer Institute, Boston, MA, USA; 4 Department of Biostatistics, Harvard School of Public Health, Boston, MA, USA; 5 Conway Institute, University College Dublin, Belfield, Dublin, Ireland

**Keywords:** colorectal cancer, fluorescence-activated cell sorting, gene expression microarray analysis, humans

## Abstract

Tumour stroma gene expression in biopsy specimens may obscure the expression of tumour parenchyma, hampering the predictive power of microarrays. We aimed to assess the utility of fluorescence-activated cell sorting (FACS) for generating cell populations for gene expression analysis and to compare the gene expression of FACS-purified tumour parenchyma to that of whole tumour biopsies. Single cell suspensions were generated from colorectal tumour biopsies and tumour parenchyma was separated using FACS. Fluorescence-activated cell sorting allowed reliable estimation and purification of cell populations, generating parenchymal purity above 90%. RNA from FACS-purified and corresponding whole tumour biopsies was hybridised to Affymetrix oligonucleotide microarrays. Whole tumour and parenchymal samples demonstrated differential gene expression, with 289 genes significantly overexpressed in the whole tumour, many of which were consistent with stromal gene expression (e.g., *COL6A3, COL1A2, POSTN, TIMP2*). Genes characteristic of colorectal carcinoma were overexpressed in the FACS-purified cells (e.g., *HOX2D* and *RHOB*). We found FACS to be a robust method for generating samples for gene expression analysis, allowing simultaneous assessment of parenchymal and stromal compartments. Gross stromal contamination may affect the interpretation of cancer gene expression microarray experiments, with implications for hypotheses generation and the stability of expression signatures used for predicting clinical outcomes.

Gene expression microarray data have huge potential for cancer research and treatment. Gene expression profiles have been used to classify tumours (Bittner *et al*, 2000; Ramaswamy *et al*, 2001; Selaru *et al*, 2002; Shipp *et al*, 2002), study the biology of tumour progression and metastasis (Birkenkamp-Demtroder *et al*, 2002; Ramaswamy *et al*, 2003), predict clinical outcomes (van de Vijver *et al*, 2002; Huang *et al*, 2003; Iizuka *et al*, 2003), classify drug resistance (Hofmann *et al*, 2002; Chang *et al*, 2003) and identify novel drug targets (Marton *et al*, 1998). As the technology matures, it is pushing towards mainstream clinical application (Gershon, 2004; Jarvis and Centola, 2005; Cardoso *et al*, 2008). Surmountable challenges remain, such as standardisation and validation of the competing platforms and analysis techniques. However, the heterogeneity of clinical tumour samples remains a fundamental problem which must be addressed (Winegarden, 2003). All cell subpopulations in a sample contribute to the gene expression profile. Relatively homogenous cell populations yield optimal expression data, and increasing ratios of stromal cells may obscure the gene expression of parenchymal cancer cells (Emmert-Buck *et al*, 1996; Ross *et al*, 2000; Butte, 2002; Sugiyama *et al*, 2002). Stromal gene expression may cause misinterpretation of data, with important subtle cancer gene changes being masked by contaminating RNA, and increasing the potential for attributing incorrect functional gene associations (Smith *et al*, 2003). However, interactions between stromal cells and tumour parenchyma are increasingly recognised as an important factor in tumour biology and clinical outcome, and the delineation and retention of stromal gene expression has considerable value (Fukino *et al*, 2007; Patocs *et al*, 2007).

Laser capture microdissection (LCM) allows the selection of specific cells from tissue and could potentially circumvent many of these problems (Emmert-Buck *et al*, 1996). Sugiyama *et al* (2002) examined the expression profile of LCM dissected tissue and whole tissue. They demonstrated significant differences in expression profiles, finding that the overall difference in the gene expression profile was related to levels of stromal contamination. However, LCM is a costly, laborious and highly skilled procedure which yields small quantities of RNA, which renders clinical application impractical (Liu, 2007). It has also been demonstrated by Michel *et al* (2003) that the LCM process introduces a systematic bias into gene expression profiles. Another problem encountered in the estimation of stromal content histologically, and therefore by both LCM and macrodissection, is the ‘reference trap’. A two-dimensional microscopic view of a complex three-dimensional structure, such as a tumour, leads to irreversible qualitative and quantitative loss of information (Nyengaard, 1999). This means that fractions of cells can be grossly under- or overestimated if unbiased sampling methods (i.e. stereological methods) are not used.

Fluorescence cytometry (FC) and fluorescence-activated cell sorting (FACS) allow simultaneous quantitation and multiparametric assessment of the phenotype of cells by staining with fluorochrome-conjugated antibodies (Afanasyeva *et al*, 2004). These are generally used to examine or sort peripheral blood samples (Waguri *et al*, 2003), to identify tumour cells in malignant effusions, to isolate clones and infrequently for the separation of specific cell populations from solid tissue (Afanasyeva *et al*, 2004; Suzuki *et al*, 2004). Advances in laser technology, speed of sorting and range of fluorochromes, make FACS a potentially useful method for identifying and purifying cell populations from solid tumours for analysis. Fluorescence-activated cell sorting overcomes many of the problems associated with LCM and macrodissection, by allowing systematic sampling of a large number of parenchymal tumour cells, allowing confirmation of the purity of targets and potentially, a better average of gene expression in cancer cells. We aimed to evaluate the feasibility of using FACS for producing homogenous cell populations for gene expression microarray analysis of colorectal tumour samples. Specifically, we wished to compare the differences in gene expression profiles elicited from whole tissue and sorted cells.

## Materials and methods

### Colorectal carcinoma tissue

Colorectal carcinoma (CRC) tissue samples were obtained, with informed consent, from patients undergoing curative bowel resection (Table 1). All patients were Irish Caucasians. None of the patients received preoperative chemotherapy or radiotherapy. Three primary cancers were used to compare the gene expression of sorted cells and whole tumour samples, after optimisation of our FACS methodology. Collection of tissue was approved by the Clinical Research Ethics Committee of the Cork Teaching Hospitals.

### Generation of a single cell suspension from colorectal cancer tissue

Tumour samples were washed in Dulbecco's modified Eagle's medium (DMEM; BioWhittaker, Wokingham, UK) and macroscopic necrotic tissue was excised with a scalpel and washed in DMEM. A portion of the biopsy was immediately snap frozen in liquid nitrogen and stored at −80°C until RNA extraction and approximately 1 g of tumour tissue was mechanically disaggregated, and subsequently enzymatically digested with bovine collagenase II, IV (Sigma-Aldrich, Dublin, Ireland) and DNAse I (Roche, Clarecastle, Ireland) at concentrations of 2 and 1 mg ml^−1^, at room temperature (22°C) for 45–60 min. This mix was then filtered through 70 mm pore mesh (Becton Dickinson, Oxford, UK). All suspensions were generated under standardised environmental conditions, being kept at 4°C, except for the enzyme digestion stage.

### Flow cytometry

Dissociated cells in suspension were incubated on ice with mouse anti-human epithelial antigen (HEA) monoclonal antibody (mAb) conjugated with FITC (clone BER-EP4; Dako, Glostrup, Denmark), anti-CD14 mAb conjugated with phycoerythrin (PE) and anti-CD45 conjugated with PerCP mAb (both from BD Pharmingen, Erembodegem-Aalst, Belgium) or relevant isotype controls, for 45 min. The labelled cells were analysed and separated using FACS Vantage with CellQuest Pro software (Becton Dickinson). Establishment of the gates was based on the staining profiles of the negative controls, positive controls (SW-620 cells, labelled with HEA) and to eliminate low forward scatter signal events, eliminating debris, red cells and apoptotic cells.

The mAb BER-EP4 binds to a partially formol-resistant epitope on the protein moiety of two 34- and 39 kDa glycopolypeptides on human epithelial cells. It does not bind to any non-epithelial cells (Latza *et al*, 1990). Specifically, it does not bind to mesenchymal or lymphoid tissue. However, in large cell populations antibodies can bind in a non-specific manner. To control for this, we blocked antibodies with 1% fetal calf serum and used isotype control antibodies as negative controls. There is also a possibility of immune cells expressing the HEA antigen after ingestion of apoptotic cells. As immune cell infiltrate is a large component of stromal tissue, we decided to use antibodies to allow us to quantify and negatively select immune cells to avoid contamination in our sorted epithelial fraction. Phagocyte numbers have been found to increase from 1.5- to 2.5-fold in Duke's B and C tumours, respectively, and T cells by 1.4-fold in colorectal tumours (Allen and Hogg, 1985, 1987). We decided that a combination of antibodies binding to CD14, which is the LPS receptor and is expressed strongly on the surface of monocytes, weakly on the surface of granulocytes and by most tissue macrophages, and CD45, a tyrosine phosphatase a critical requirement for T- and B-cell antigen receptor-mediated activation, which is expressed, typically at high levels, on all haematopoietic cells (expression is at a higher density on lymphocytes, approximately 10% of surface area is CD45), would be the ideal combination. It has previously been demonstrated that using positive selection of HEA-expressing cells and negative selection of CD45- or CD14-expressing cells yields using immunomagnetic cell sorting lead to high yields of epithelial cells from cell solutions (Zigeuner *et al*, 2000; Guo *et al*, 2004).

### Cell sorting and confirmation of cell phenotype

**Figure 1:**
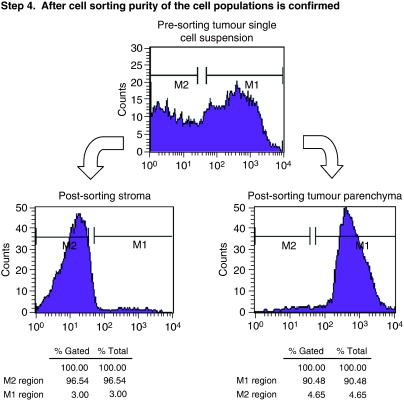
This figure demonstrates our method for separating tumour stroma and parenchymal cells using fluorescence-activated cell sorting. Briefly, debris is gated out, target populations identified and positively (parenchyma) or negatively selected (stroma). Dot plots represent 10 000 events, and show side scatter and forward scatter plots in Step 1 and fluorescence plots in Steps 2 and 3. Cells have been simultaneously stained with mouse anti-human anti-HEA, anti-CD14 and anti-CD45 monoclonal antibodies conjugated with FITC, PE and PerCP, respectively, which are resolved on FL-1, FL-2 and FL-3. Step 1 demonstrates our method of gating out debris and identification of the HEA positive population with scatter plots. Step 2 demonstrates the identification of CD45 (A) and CD14 (B) positive populations, followed by gating of HEA+CD14 population (C) and gating of HEA+ CD14-CD45-population (D), which consists of 43% of cells. Step 3 is the identification and selection of the R3 region (HEA+ CD14- CD45- population) for cell sorting, and the estimation of presorting parenchymal content, which is estimated at only 43% of cells in this sample. After cell sorting the flow cytometer is cleaned, and the post-sorting populations evaluated. Step 4 shows a histogram demonstrating the post-sorting populations of cells. The histogram plots the fluorescence of HEA^+^ cells on FL-1 (region M2) against counts on the *y*-axis. There is clear separation of the tumour parenchyma (M2 region) and the stromal (M1 region) cells. We estimate the populations have a purity of over 90%.

A one-step, three-colour, sorting approach was used. Our goal was to positively select colorectal parenchyma and negatively select for stromal cells. A diagram illustrating the method is shown in Figure 1. Sorting gates were set for positive selection of HEA^+^ CD14^−^ CD45^−^ and negative selection of HEA^−^ CD14^+^ CD45^+^ cells. Unstained cells, cells stained with isotype controls, were used for all samples, and SW-620 cells were used as a positive control for CRC cells. At least 7 million HEA^+^ cells were sorted, as below this level we found RNA quantity was variable. Cells were sorted into BD polypropylene flow tubes coated with 4% bovine serum albumin (Sigma-Aldrich). Purity of the sample was checked after sorting by reanalysing HEA^+^ CD14^−^ CD45^−^ fraction, on the same machine, after a full cleaning protocol. Purity greater than 90% was deemed acceptable.

After sorting, cells were confirmed to be colorectal tumour cells by microscopic examination, by cytospinning on to Superfrost Plus microscope slides (BDH Laboratory Supplies, Poole, UK) followed by ethanol fixation and staining with Rapi-Diff (Cytocolor, Hinckley, OH, USA) or immunocytochemistry. Immunocytochemistry was performed using Dako MNF-116 anti-pan-cytokeratin antibody, using the standard EnVision kit protocol (Dako).

We have also applied this method to successfully purify tumour parenchyma in CRC liver metastases, primary breast tumours, and with modification, to sort breast cancer bone marrow micrometastases.

### RNA isolation

RNA was extracted from three corresponding whole tumour samples and FACS-purified tumour parenchyma using a modification of the Tri Reagent (Molecular Research Center, Cincinnati, OH, USA) protocol (Curtin and Cotter, 2004). RNA with an absorbance ratio A260/240 >1.8 and no evidence of RNA degradation by gel electrophoresis was accepted. We then checked the RNA quality using the Agilent Bioanalyzer (Agilent, Santa Clara, CA, USA) runs. We used RNA with a RIN (RNA integrity number) value ⩾8 (Schroeder *et al*, 2006).

### cRNA preparation

The labelling of the total RNA was performed according to the ‘Small Sample Labeling Protocol vII’ (Affymetrix, Santa Clara, CA, USA). Total RNA (100 ng) was used as starting material for the first round of cDNA preparation. The first and second strand cDNA synthesis was performed using the Superscript II system (Invitrogen, Dublin, Ireland) according to the manufacturer's instructions except using an oligo-dT primer containing a T7 RNA polymerase promoter site. The first round of *in vitro* transcription (IVT) was performed using the MEGAscript T7 kit (Ambion, Warrington, UK). The second round of cDNA preparation was done as first round except now random hexamers replaced the oligo-dT primer.

Labelled cRNA was prepared using the BioArray High Yield RNA Transcript Labeling Kit (Enzo, Farmingdale, NY, USA). Biotin labelled CTP and UTP (Enzo) were used in the reaction together with unlabelled NTPs. During the labelling, the IVT product and also the fragmented IVT product were checked by gel electrophoresis. Following the IVT reaction, the unincorporated nucleotides were removed using RNeasy columns (Qiagen, Crawley, UK).

### Oligonucleotide array hybridisation and scanning

Fragmented cRNA was loaded onto the GeneChip HU133 Plus 2.0 probe array cartridge (Affymetrix). The washing and staining procedure was performed in the Affymetrix Fluidics Station 450 (Affymetrix). The biotinylated cRNA was stained with a streptavidin–PE conjugate, and the probe arrays were scanned at 560 nm using a confocal laser-scanning microscope (Affymetrix Scanner 3000; Affymetrix). After hybridisation and scanning, we checked several quality parameters: scaling factor ⩽3-fold difference within a study; 3′/5′ ratio for probe sets for GAPDH ⩽3; present (P) calls in the same range for all samples in the study and RawQ below 100. All of our arrays passed all stages of the quality control. The readings from the quantitative scanning were analysed by the Affymetrix Gene Expression Analysis Software (Affymetrix).

### Statistical analysis of gene expression data

Affymetrix GeneChip array data were normalised, pre-processed and analysed using R and Bioconductor statistical software (Gentleman *et al*, 2004). Raw CEL file data from human whole genome Affymetrix U133Plus2 gene GeneChips of purified ‘of whole primary’ colon cancer samples (*n*=3) and tumour parenchyma samples purified by FACS (*n*=3) were imported into R. Initial exploratory data analysis performed using the overview function in the package made4 (Culhane *et al*, 2005) suggested that the assumption of a constant sum across all microarray samples may not be valid for these data. Moreover, there were significantly more MAS 5.0 P calls in the whole samples than in the FACS-purified samples (paired *t*-test, *P*<0.05). Therefore, data were normalised using the Li and Wong's invariant set method using the ‘expresso’ function in the Affy package in Bioconductor (Li and Hung Wong, 2001; Gentleman *et al*, 2004). Normalised data were log2 transformed and assessed initially using use two exploratory data analysis approaches: hierarchical cluster analysis (1-Pearson correlation coefficient distance with average linkage joining) and dimension reduction using correspondence analysis (COA; Eisen *et al*, 1998; Fellenberg *et al*, 2001). Figures were created using the made4 package in Bioconductor (Culhane *et al*, 2005).

### Detection of genes differentially expressed in purified tumour

Given the low number of replicates in this study, it is challenging to estimate of gene mean and variance; therefore, rank-based non-parametric methods may be more efficient in these data. It is reported that rank product performs comparably or outperforms *t*-statistic-based methods when replicates numbers are very low (less than five) (Breitling *et al*, 2004; Jeffery *et al*, 2006). Rank products analysis is a non-parametric statistic that detects genes that are consistently highly ranked in lists, that is genes that are consistently upregulated genes in a number of replicate experiments. Rank products analysis does not require a measure of gene-specific variance and is therefore particularly powerful when only a small number of replicates are available. Rank products analysis was performed using the Bioconductor package RankProd. False discovery rates were estimated using 100 permutations.

To aid interpretation of these genes lists, we used DAVID to assess which Gene Ontology biological and functional categories were overrepresented in this list of genes (Dennis *et al*, 2003). We used the highest stringency level, for other analyses we used an EASE of 0.01, and false discovery rate of 1000. Heatmap images of gene expression profiles were generated using the made4 package in Bioconductor. The Human Genome Organisation (HUGO) gene symbols for Affymetrix probe sets were retrieved using the annaffy Bioconductor package and the annotation library hu133plus2 (build Tuesday, 4 October 2005, 20:53:27).

## Results

### Patient demographics and FACS

**Figure 2:**
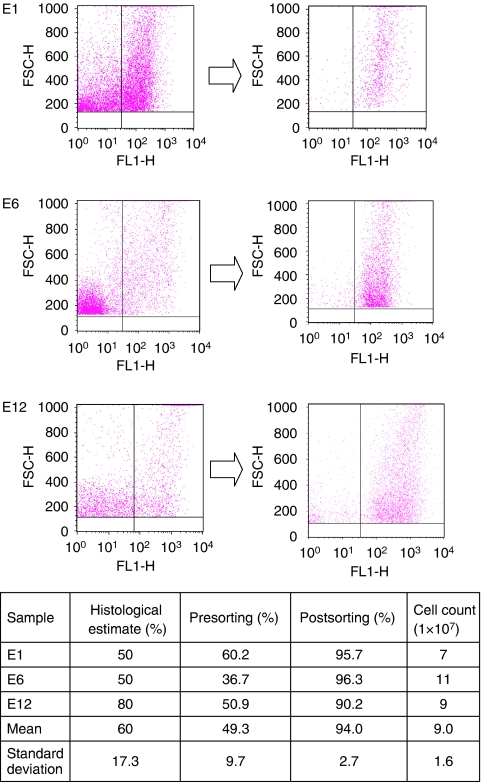
Fluorescence-activated cell sorting of CRC cells from experimental samples. We generated single cell suspensions from three patient samples and sorted them to greater than 90% purity in each case. Dot plots show the pre- and post-sorting HEA^+^ populations. The table shows the estimates of tumour cell purity in the samples and the initial histological estimates of tumour parenchyma content. We sorted an average of 9 million cells per sample.

**Figure 3:**
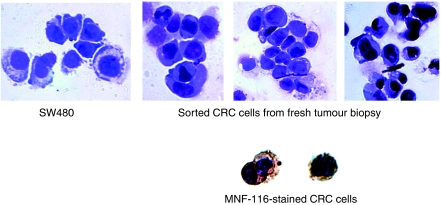
Cytological confirmation of cell-sorted tumour parenchyma. To confirm that our HEA^+^ CD14^−^ CD45^−^ cell population was tumour parenchyma, we examined three post-sorting populations after cytospinning and Rapi-Diff II (Diagnostic Developments) staining and compared them to SW-480 cells, a cell line derived from a primary colorectal tumour ( × 40 magnification shown). Then we confirmed the cells were epithelial by staining with MNF-116 pan-cytokeratin (Dako).

Three matched patient biopsies were taken immediately after resection. No patients received pre-operative chemoradiotherapy. Metastases (m1) were observed intra-operatively in one patient. The other patients were free from metastases (mx). Histological examination of the E1 and E12 samples demonstrated moderately differentiated adenocarcinomas with strand-like infiltrative pattern of malignant glands through muscularis propria, interspersed by stroma. The E6 sample demonstrated a well-differentiated adenocarcinoma with closely packed glands invading into muscularis propria, with stroma between the glands. After generation of single cell suspensions from our experimental tumour biopsies, stromal content was estimated to range from 37 to 60%, and sorted to greater than 90% purity as described earlier (Figure 2). The parenchymal component of the tumours was estimated to range from 50 to 80% on histological assessment. The E6 sample demonstrated the biggest discrepancy in estimation of parenchymal content (FC estimate 37 *vs* 80% histological assessment), which may be explained by the fact that the sample contained a large muscularis propria component, which could account for the high proportion of non-staining FC events. Sorted cells were subsequently confirmed as tumour parenchyma by light microscopy assessment (Figure 3). We found the sorted cell population was homogenous and had the morphological appearances consistent with CRC cells after staining with Rapi-Diff and comparison to SW-620 colorectal cell line. The cells also stained positive for the cytokeratin MNF-116, confirming they were epithelial in nature. Using FC we found that the population of HEA^+^ cells fluoresced in the same region as cells stained with MNF-116.

### Differential gene expression

**Figure 4:**
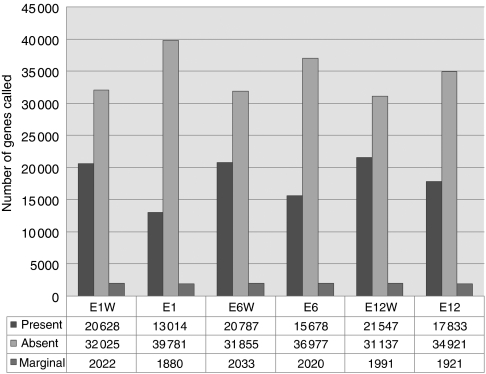
Differential gene expression between whole tumour and cell-sorted colorectal cancer biopsies. This graph displays the genes called as presented by MAS 5.0 in each sample (P, present; A, absent; M, marginal). There was a significant difference in genes called as present in the whole-tissue samples (*P*=0.0407, Student's paired *t*-test). These genes are therefore expressed in non-parenchymal (or stromal) tissue.

**Figure 5:**
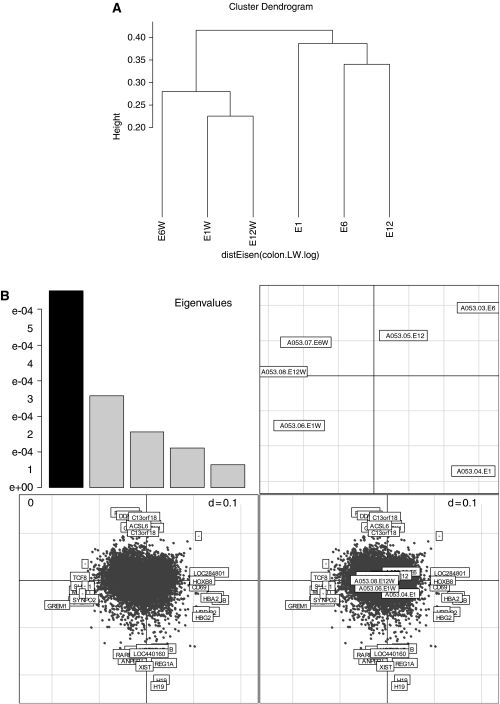
Hierarchical clustering using Eisen's formula of a correlation similarity metric and average linkage clustering led to the cell-sorted samples and whole-tissue samples clustering together, showing they are most similar to each other (**A**). This is despite the fact that they are paired samples. Correspondence analysis also demonstrates that the sorted samples and whole-tissue samples cluster together (**B**). The first axis (horizontal) splits the whole and FACS-purified samples. The second axis (vertical) split E1 and E1W from E6, E12, E12W and E6W. Genes that separate the samples are shown with HUGO classification.

There were significantly more MAS 5.0 P calls in the whole samples than in the FACS-purified samples (paired *t-*test, *P*<0.05; Figure 4). Ordination was used to explore the data, and correspondence analysis was applied to the data using the made4 package in Bioconductor (Culhane *et al*, 2005). Correspondence analysis is a useful dimension reduction method for observing the *χ*^2^ or associations between genes and samples. The dendrogram showed that the whole and the purified samples could be portioned into two distinct clusters (Figure 5A). These clusters were also observed on the most variant or first axis of a COA of these data (Fellenberg *et al*, 2001) (Figure 5B). Interestingly, the second most variant axis (F2, vertical) separated the metastatic (E1) and metastatic-free samples. Although we have few replicates in this study, it appeared that metastatic and metastatic-free tumour samples were more defined in the purified samples when compared to the whole samples. The discrimination between metastatic and metastatic-free samples accounted for more variance than difference between tumour stage.

**Figure 6:**
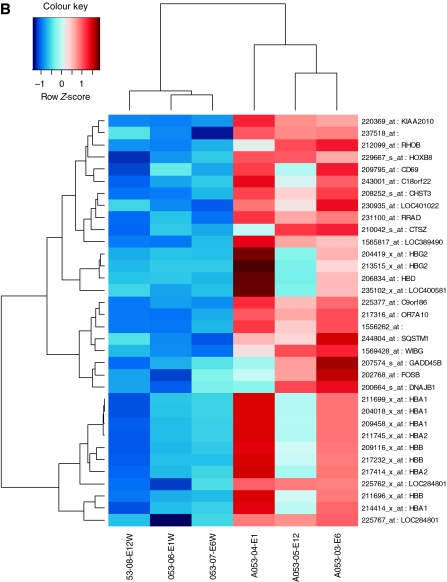
Genes detected by rank products analysis (*P*<0.01). Heatmaps showing the differentially expressed genes detected in whole but not purified samples (**A**) and purified but not whole samples (**B**) using rank product analysis (*P*<0.01) using *Z*-score normalised values (row centred). Red tiles represent upregulated genes and blue represent downregulated genes.

Expression of 289 genes were detected in whole-biopsy samples but not in purified samples (*P*<0.05). Of these, 50 differentially expressed genes were highly significant (*P*<0.01; Table 2). Expression of 103 genes were detected in purified samples, but significantly downregulated in whole samples (*P*<0.05), of which 33 of these were significant at *P*<0.01 (Table 3) which are displayed in the Heatmaps in. Heatmaps of the highly significant differentially expressed genes were generated, and displayed in Figure 6.

### Functional annotation

We used DAVID to classify gene function in the whole tumour sample (Table 4). Most functional classes are consistent with stromal as opposed to tumour cell function (e.g., proteinaceous extracellular matrix *P*=2.37 × 10^−08^, extracellular matrix *P*=2.49 × 10^−08^, collagen triple helix repeat *P*=7.12 × 10^−07^), although genes known to be expressed in tumour epithelium were identified (e.g., *GREM1*). Looking at individual genes, upregulation of connective tissue genes was prevalent (e.g., *COL6A3, COL1A2, COL12A1, COL5A2, COL3A1, CTHRC1, SULF1*), as were genes involved in extracellular matrix function (e.g., *LAMA4, PI3, POSTN, TIMP2*) and cell adhesion (e.g., *TNS1*). Genes involved in endothelial function (e.g., *TIMP2*) and specifically, colon cancer tumour endothelium, such as the anthrax toxin receptor (*ANTXR1*), were also upregulated (Liu *et al*, 2008).

In contrast, the 31 highly significantly expressed genes in the FACS-purified cells do not display characteristics of stromal gene expression, and may be representative of tumour parenchyma gene expression. Genes involved in cell signalling, such as *SQSTM1*, which regulates activation of the nuclear factor-*κ*B (NF-*κ*B) signalling pathway, receptor internalisation, and protein turnover, and *RRAD*, a member of the Ras/GTPase superfamily, are also overexpressed (Moyers *et al*, 1997; Seibenhener *et al*, 2007). Genes known to be expressed in CRC were also significantly upregulated, such as *HOX2D* and *RHOB*, which mediate apoptosis in neoplastic cells, and are targets for novel antitumour agents, such as farnesyltransferase inhibitors (Vider *et al*, 1997; Delarue *et al*, 2007).

### Comparison with Kwong *et al* expression signature

Kwong *et al* (2005) examined the expression signature derived from 60 tumours (normal mucosa, adenoma, tumour and liver metastases) and identified an expression profile that was able to differentiate between normal and neoplastic samples, but not individual tumour stages. They suggested that stromal genes may obscure the subtle molecular changes in tumours of differing pathological stage. Examination of their gene list, specifically the 34 upregulated genes in their signature, reveals the expression of extracellular matrix proteins such as, collagen, type I, *α*1 (*COL1A1*) and fibronectin. The authors believe this represented gene expression derived from infiltrating lymphocytes and other stroma. The gene list they identified shares similarities to gene expressed in our whole tumour sample (*ANTXR1, COL12A1, COL5A2, CTHRC1, POSTN*).

## Discussion

Our study demonstrates that it is feasible to reproducibly separate and purify tumour parenchyma and other cell populations from a single cell suspension generated from a solid tumour using FACS. We also found that the gene expression profile elicited from the whole tumour was significantly different from that of the purified tumour parenchyma, and that source of this differential expression may be tumour stroma. When tumour parenchymal purity is necessary, FACS may be an alternative to LCM, in particular in tumours such as CRC, melanoma and other non-sclerotic tumours amenable to the generation of a single cell suspension.

In our samples, we noted a large variance in estimated quantity of tumour parenchyma and stroma. Using FC we estimated that the parenchymal component of the tumours ranged from 37 to 60% and stroma from 40 to 63%. This was enriched to over 90% (range 90–96%) in each case with one sorting run per sample after calibration of the machine and settings for each sample. Verification of cell type is straightforward with standard staining techniques. We believe this level of stroma would grossly affect the gene expression profile taken from the biopsy and would be in keeping with the findings of Sugiyama *et al* (2002).

For publication we specifically used rank products as this has been shown to be reliable in small sample sizes in microarray experiments (Breitling *et al*, 2004; Jeffery *et al*, 2006). We found that the expression profile in the whole elicited also made biological sense. We are confident that there are real differences in the gene identified, which are related to the stromal gene expression, and that this has implications for clinical application of gene expression microarrays in CRC.

The MammaPrint assay, which is a clinical application of the van‘t Veer 70-gene breast cancer expression profile, relies on a single fresh sample of tumour to predict prognosis (van ‘t Veer *et al*, 2002; Cardoso *et al*, 2008). The samples are examined, and a stromal content of <50% is deemed acceptable for the test. This would arguably eliminate two of our samples from analysis, which we were able to enrich to >90% purity. However, Wang *et al* (2004), who derived an expression profile predicting recurrence of Duke's B colorectal carcinoma, included only samples that were enriched to over 85% purity. We believe that in CRC samples, the stroma will contaminate the sample causing problems with patient classification, but that can be overcome with parenchymal purification. The optimal tumour/stroma ratio for gene expression studies is yet to be determined and may vary depending on the tumour type.

To ensure good quality expression data, we used several layers of quality control, starting with RNA gel electrophoresis and then checking RNA integrity with the Agilent Bioanalyzer (Agilent). Subsequently, after hybridisation and scanning, we checked several quality parameters such as scaling factor, 3′/5′ ratios, P calls and RawQ values. Quantitative RT–PCR was not employed as all of our arrays passed all stages of the quality control, and we do not believe that it will be used in clinical practice. This has been borne out by the current application of the MammaPrint assay and is a similar approach to other clinical microarray studies (Wang *et al*, 2004; Glas *et al*, 2006; Ach *et al*, 2007; Cardoso *et al*, 2008).

We found a significant difference in the total number of probes called as present using the Affymetrix MAS5 signal algorithm. This shows that the whole tissue expressed a larger number of genes than sorted cells, demonstrating the wide range of genes expressed by non-tumour cells. We used Li and Wong's invariant set method for normalisation of the data sets (Li and Hung Wong, 2001). This uses a set of non-differentially expressed genes to normalise data that are identified by using an iterative procedure. Gene expression common to the sorted cells and whole tissue should be reasonably uniform. Any genes found to be expressed in the whole tissue can be presumed to be contained in stromal tissue. We then sought to examine how similar the gene expression profile of each sample was. Using hierarchical clustering, all three sorted samples clustered together, as did the whole-tissue samples clustered together. Correspondence analysis also showed a clear separation of sorted samples and whole-tissue samples. The E1 and E1W sample also separated from the other samples. This is not surprising as this sample was from a more advanced tumour than the others. The stromal component of the whole-tissue sample was the biggest determinate in differences and could easily separate all samples. This may explain why some studies show very similar GEP throughout tumour stages, and the similarity of some of the genes in our whole tumour sample to that of Kwong *et al* (Birkenkamp-Demtroder *et al*, 2002; Kwong *et al*, 2005). Qualitatively the expression of the whole tumour samples were consistent with tumour stroma, with genes highly specific for colorectal tumour stroma (e.g., *ANTXR1*), and DAVID analysis identifying highly significant functional groups involved in extracellular matrix function. Conversely, FACS-purified parenchyma expressed genes specifically associated with colorectal neoplasia, such as *HOX2D* and *RHOB*. The ability to examine the samples in parallel affords increased precision in analysis of tumour fraction gene expression and offers new opportunities to examine tumour–stroma interactions.

Fluorescence-activated cell sorting parenchymal purification has several advantages over LCM. Disaggregation of a tumour sample generates a large random sample of tumour cells and may elicit a more representative and relevant gene expression profile than LCM, without the need for RNA amplification. Previous studies have assessed the differences between LCM acquired tissue, macrodissection and whole-tissue samples for microarray studies (Sugiyama *et al*, 2002; Michel *et al*, 2003; de Bruin *et al*, 2005). Sugiyama suggests that if the stromal compartment is >30% LCM should be used, and showed marked differences in GEP in LCM-derived tissue compared with bulk biopsy. Similarly, Michel *et al* (2003) demonstrated that the LCM process introduces a bias into GEP profiles. Although they found that large expression changes were maintained, many genes changed with lower expression levels may be lost. This is problematic for several reasons – particularly as smaller changes in mRNA expression may have larger effects downstream than larger ones. Also it makes comparisons difficult between studies. Although LCM aims to overcome such problems, the very premise it is built on may introduce bias. A two-dimensional microscope view of a complex three-dimensional structure such as a tumour leads to irreversible qualitative and quantitative loss of information (Nyengaard, 1999). This means that fractions of cells can be grossly under- or overestimated if unbiased sampling methods such as stereological methods are not used (the ‘reference trap’). At worst it can lead to a gene expression profile of a tiny fraction of tumour being misinterpreted as expression of the whole tumour. Macrodissection is subject to similar compromises. Fluorescence-activated cell sorting overcomes many of these problems by allowing systematic sampling of cells, providing a large sample of cells, which allows confirmation of purity of targets and also a better average of gene expression in a tumour.

In conclusion, FACS is effective in producing homogenous cell populations for gene expression microarray experiments in solid tumours and is viable alternative to macrodissection, LCM and whole tumour sampling in microarray experiments. Fluorescence-activated cell sorting overcomes many of the practical and theoretical problems associated with LCM. The gene expression profile of FACS-purified tumour parenchyma is significantly different to that of clinically resected tumour biopsies. Our analysis suggests that stromal gene expression is responsible for the differential expression and makes a significant contribution to the gene expression profile of whole tumour CRC biopsies. Therefore, one should consider a purification strategy when planning solid tumour gene expression microarray experiments. Although many of the sources of technical noise and variation in gene expression microarray technology have been overcome, there remain challenges, such as the approach to tumour heterogeneity, which need to be overcome before it is accepted into clinical practice.

## Figures and Tables

**Table 1:** Microarray sample details

**CEL file name**	**Patient**	**Sample type**	**TNM classification**	**Stage**	**Location**	**Sex**	**Age (years)**
A053-04-E1	E1	FACS	T4N0M1	IV	Caecum	Female	68
A053-03-E6	E6	FACS	T2N0Mx	I	Rectosigmoid	Male	55
A053-05-E12	E12	FACS	T3N0Mx	IIA	Sigmoid	Male	35
A053-06-E1W	E1	Whole tissue	T4N0M1	IV	Caecum	Female	68
A053-07-E6W	E6	Whole tissue	T2N0Mx	I	Rectosigmoid	Male	55
A053-08-E12W	E12	Whole tissue	T3N0Mx	IIA	Sigmoid	Male	35

**Table 2:** Genes called significant in whole tumour (*P*<0.01, rank products)

**Probe set ID**	**Gene symbol**	**Gene title**	**RP/Rsum**	**pfp**
207961_x_at	*MYH11*	Myosin, heavy chain 11, smooth muscle	53.331	0
218469_at	*GREM1*	Gremlin 1, cysteine knot superfamily, homolog (*Xenopus laevis*)	18.3444	0
205594_at	*ZNF652*	Zinc-finger protein 652	123.6405	0.0011
226663_at	*ANKRD10*	Ankyrin repeat domain 10	122.7271	0.0013
201438_at	*COL6A3*	Collagen, type VI, *α*3	113.3836	0.0014
212764_at	*ZEB1*	Zinc finger E-box-binding homeobox 1	139.7127	0.0015
224823_at	*MYLK*	Myosin light chain kinase	108.5523	0.0017
228030_at	—	Transcribed locus, strongly similar to NP_005768.1 RNA-binding motif protein 6 [*Homo sapiens*]	138.618	0.0017
212077_at	*CALD1*	Caldesmon 1	134.6639	0.0018
206199_at	*CEACAM7*	Carcinoembryonic antigen-related cell adhesion molecule 7	133.868	0.002
212354_at	*SULF1*	Sulfatase 1	93.7657	0.002
235028_at	—	CDNA FLJ42313 fis, clone TRACH2019425	70.702	0.0025
230269_at	—	Transcribed locus	145.6395	0.0029
241879_at	—	Transcribed locus	64.2024	0.0033
227260_at	*ANKRD10*	Ankyrin repeat domain 10	201.5746	0.0064
227061_at	—	CDNA FLJ44429 fis, clone UTERU2015653	209.8229	0.0066
202202_s_at	*LAMA4*	Laminin, *α*4	200.6896	0.0067
218468_s_at	*GREM1*	Gremlin 1, cysteine knot superfamily, homolog (*Xenopus laevis*)	194.7291	0.0067
238750_at	—	Transcribed locus	211.7612	0.0067
225269_s_at	*C2orf12*	Chromosome 2 open-reading frame 12	183.4678	0.0068
227140_at	—	CDNA FLJ11041 fis, clone PLACE1004405	197.6319	0.0068
203691_at	*PI3*	Peptidase inhibitor 3, skin-derived (SKALP)	200.1168	0.0069
208747_s_at	*C1S*	Complement component 1, s subcomponent	194.1383	0.007
221748_s_at	*TNS1*	Tensin 1	185.7447	0.007
225681_at	*CTHRC1*	Collagen triple helix repeat containing 1	215.9928	0.0071
1557270_at	—	CDNA FLJ36375 fis, clone THYMU2008226	171.7643	0.0072
202404_s_at	*COL1A2*	Collagen, type I, *α*2	191.9636	0.0073
225786_at	*LOC284702*	Hypothetical protein LOC284702	162.5829	0.0073
225664_at	*COL12A1*	Collagen, type XII, *α*1	166.478	0.0075
243134_at	—	Transcribed locus	219.3881	0.0075
210809_s_at	*POSTN*	Periostin, osteoblast-specific factor	188.6781	0.0076
225275_at	*EDIL3*	EGF-like repeats and discoidin I-like domains 3	171.1717	0.0076
221729_at	*COL5A2*	Collagen, type V, *α*2	231.1445	0.0086
202948_at	*IL1R1*	Interleukin 1 receptor, type I	252.7068	0.0087
225107_at	*HNRNPA2B1*	Heterogeneous nuclear ribonucleoprotein A2/B1	243.1025	0.0087
226731_at	*PELO*	Pelota homolog (*Drosophila*)	246.3912	0.0087
209656_s_at	*TMEM47*	Transmembrane protein 47	230.2568	0.0088
218353_at	*RGS5*	Regulator of G-protein signaling 5	254.393	0.0088
224565_at	*TncRNA*	Trophoblast-derived noncoding RNA	273.7687	0.0088
212067_s_at	*C1R*	Complement component 1, r subcomponent	241.6854	0.0089
201852_x_at	*COL3A1*	Collagen, type III, *α*1 (Ehlers-Danlos syndrome type IV, autosomal dominant)	271.8757	0.009
208782_at	*FSTL1*	Follistatin-like 1	268.8627	0.009
212353_at	*SULF1*	Sulfatase 1	265.4504	0.0091
221778_at	*JHDM1D*	Jumonji C domain containing histone demethylase 1 homolog D (*S. cerevisiae*)	229.742	0.0091
224694_at	*ANTXR1*	Anthrax toxin receptor 1	268.3533	0.0091
225809_at	*DKFZP564O0823*	DKFZP564O0823 protein	261.4881	0.0091
215076_s_at	*COL3A1*	Collagen, type III, *α*1 (Ehlers-Danlos syndrome type IV, autosomal dominant)	240.3341	0.0092
1555878_at	*RPS24*	Ribosomal protein S24	260.4235	0.0093
201540_at	*FHL1*	Four-and-a-half LIM domains 1	258.0377	0.0093
231579_s_at	*TIMP2*	TIMP metallopeptidase inhibitor 2	265.3035	0.0093

**Table 3:** Genes called significant in FACS purified CRC cells (*P*<0.01, rank products)

**Probe set ID**	**Gene symbol**	**Gene title**	**RP/Rsum**	**pfp**
200664_s_at	*DNAJB1*	DnaJ (Hsp40) homolog, subfamily B, member 1	68.0709	0
204018_x_at	*HBA1*	Haemoglobin, *α*1	66.0374	0
209116_x_at	*HBB*	Haemoglobin, *β*	10.5524	0
209458_x_at	*HBA1*	Haemoglobin, *α*1	60.4785	0
211699_x_at	*HBA1*	Haemoglobin, *α*1	67.0989	0
211745_x_at	*HBA1*	Haemoglobin, *α*1	52.9371	0
214414_x_at	*HBA1*	Haemoglobin, *α*1	61.1365	0
217232_x_at	*HBB*	Haemoglobin, *β*	8.1213	0
217316_at	*OR7A10*	Olfactory receptor, family 7, subfamily A, member 10	59.4448	0
217414_x_at	*HBA1*	Hemoglobin, *α*1	28.7839	0
225762_x_at	*LOC284801*	Hypothetical protein LOC284801	9.059	0
225767_at	—	—	42.7413	0
229667_s_at	*HOXB8*	Homeobox B8	34.4176	0
204419_x_at	*HBG1*	Haemoglobin, *γ*A	97.798	0.0012
1565817_at	*IKZF1*	IKAROS family zinc-finger 1 (Ikaros)	93.4767	0.0013
211696_x_at	*HBB*	Haemoglobin, *β*	92.8953	0.0013
209795_at	*CD69*	CD69 molecule	92.588	0.0014
231100_at	*RRAD*	Ras-related associated with diabetes	135.6185	0.0044
207574_s_at	*GADD45B*	Growth arrest and DNA-damage-inducible, *β*	152.1069	0.0052
208252_s_at	*CHST3*	Carbohydrate (chondroitin 6) sulfotransferase 3	149.7873	0.0052
212099_at	*RHOB*	Ras homolog gene family, member B	151.4472	0.0055
225377_at	*C9orf86*	Chromosome 9 open-reading frame 86	145.8861	0.0055
230935_at	—	—	144.722	0.0058
243001_at	*C18orf22*	Chromosome 18 open-reading frame 22	167.4874	0.006
202768_at	*FOSB*	FBJ murine osteosarcoma viral oncogene homolog B	163.3693	0.0062
210042_s_at	*CTSZ*	Cathepsin Z	174.6238	0.0062
1569428_at	*WIBG*	Within bgcn homolog (*Drosophila*)	190.2299	0.0071
206834_at	*HBB*	Haemoglobin, *β*	203.1012	0.0073
1556262_at	—	CDNA clone IMAGE:4822139	208.8925	0.0074
220369_at	*SMEK1*	SMEK homolog 1, suppressor of mek1 (*Dictyostelium*)	189.2357	0.0074
244804_at	*SQSTM1*	Sequestosome 1	210.7027	0.0075
235102_x_at	—	Transcribed locus	199.6653	0.0076
213515_x_at	*HBG1*	Haemoglobin, *γ*A	223.858	0.0088
237518_at	—	Transcribed locus	225.91	0.0091

**Table 4:** Functional classification of whole tumour gene expression with DAVID (top 50)

**Category**	**Term**	**Gene count**	**Percentage**	***P*-value**
GO_CC	Extracellular region part	15	32.61%	3.87E−10
GO_CC	Extracellular region	17	36.96	6.31E−10
SP_PIR	Hydroxylation	7	15.22	5.82E−09
GO_CC	Proteinaceous extracellular matrix	10	21.74	2.37E−08
SP_PIR	Extracellular matrix	9	19.57	2.49E−08
GO_CC	Extracellular matrix	10	21.74	2.71E−08
SP_PIR	Signal	22	47.83	3.88E−08
GO_CC	Extracellular matrix part	7	15.22	1.00E−07
UP_SEQ	Signal peptide	22	47.83	1.49E−07
SP_PIR	Trimer	5	10.87	3.61E−07
INTERPRO	Collagen triple helix repeat	6	13.04	7.12E−07
SP_PIR	Triple helix	5	10.87	7.53E−07
SP_PIR	Hydroxylysine	5	10.87	8.60E−07
SP_PIR	Secreted	15	32.61	8.62E−07
GO_BP	Phosphate transport	6	13.04	9.47E−07
SP_PIR	Hydroxyproline	5	10.87	1.10E−06
GO_CC	Collagen	5	10.87	1.22E−06
SP_PIR	Collagen	6	13.04	1.24E−06
SP_PIR	Direct protein sequencing	18	39.13	2.48E−06
SP_PIR	Pyroglutamic acid	5	10.87	3.81E−06
SP_PIR	Structural protein	6	13.04	6.91E−06
INTERPRO	Collagen helix repeat	5	10.87	7.04E−06
GO_BP	Organic anion transport	6	13.04	1.73E−05
GO_BP	Anion transport	6	13.04	3.99E−05
GO_MF	Extracellular matrix structural constituent	5	10.87	6.54E−05
KEGG	ECM-receptor interaction	5	10.87	7.67E−05
UP_SEQ	Short sequence motif:Cell attachment site	5	10.87	9.21E−05
PIR_SUPERFAMILY	Collagen *α*1(I) chain	3	6.52	9.79E−05
KEGG	Focal adhesion	6	13.04	1.39E−04
SP_PIR	Glycoprotein	19	41.30	1.57E−04
GO_BP	System development	13	28.26	1.62E−04
GO_MF	Structural molecule activity	9	19.57	1.68E−04
UP_SEQ	Propeptide:C-terminal propeptide	3	6.52	1.83E−04
SP_PIR	Ehlers-Danlos syndrome	3	6.52	1.89E−04
GO_BP	Organ development	11	23.91	2.43E−04
GO_BP	Extracellular matrix organization and biogenesis	4	8.70	2.45E−04
GO_CC	Fibrillar collagen	3	6.52	2.71E−04
INTERPRO	Fibrillar collagen, C-terminal	3	6.52	2.84E−04
KEGG	Cell communication	5	10.87	4.06E−04
GO_BP	Multicellular organismal development	14	30.43	5.90E−04
GO_BP	Multicellular organismal process	17	36.96	6.33E−04
UP_SEQ	Disulphide bond	15	32.61	6.92E−04
SMART	COLFI	3	6.52	7.14E−04
UP_SEQ	Glycosylation site:N-linked (GlcNAc…)	18	39.13	8.15E−04
GO_BP	Anatomical structure development	13	28.26	0.001069024
UP_SEQ	Domain:VWFC	3	6.52	0.001162772
GO_BP	Extracellular structure organization and biogenesis	4	8.70	0.001291298
INTERPRO	von Willebrand factor, type C	3	6.52	0.003487936
GO_BP	Developmental process	15	32.61	0.004413792
GO_MF	Complement component C1s activity	2	4.35	0.004676533
GO_CC	Extracellular space	6	13.04	0.005323916
UP_SEQ	Domain:VWFA 4	2	4.35	0.006011568
SMART	VWC	3	6.52	0.007203275
SP_PIR	Pyrrolidone carboxylic acid	3	6.52	0.008914529
SP_PIR	Skin	2	4.35	0.009320842

## References

[bib1] Ach RA, Floore A, Curry B, Lazar V, Glas AM, Pover R, Tsalenko A, Ripoche H, Cardoso F, d’Assignies MS, Bruhn L, Van't Veer LJ (2007) Robust interlaboratory reproducibility of a gene expression signature measurement consistent with the needs of a new generation of diagnostic tools. BMC Genomics 8: 148 17553173 10.1186/1471-2164-8-148 PMC1904205

[bib2] Afanasyeva M, Georgakopoulos D, Belardi DF, Ramsundar AC, Barin JG, Kass DA, Rose NR (2004) Quantitative analysis of myocardial inflammation by flow cytometry in murine autoimmune myocarditis: correlation with cardiac function. Am J Pathol 164: 807–815 14982835 10.1016/S0002-9440(10)63169-0 PMC1613271

[bib3] Allen C, Hogg N (1985) Monocytes and other infiltrating cells in human colorectal tumours identified by monoclonal antibodies. Immunology 55: 289–299 3891596 PMC1453624

[bib4] Allen C, Hogg N (1987) Elevation of infiltrating mononuclear phagocytes in human colorectal tumors. J Natl Cancer Inst 78: 465–470 3546908

[bib5] Birkenkamp-Demtroder K, Christensen LL, Olesen SH, Frederiksen CM, Laiho P, Aaltonen LA, Laurberg S, Sorensen FB, Hagemann R, TF OR (2002) Gene expression in colorectal cancer. Cancer Res 62: 4352–4363 12154040

[bib6] Bittner M, Meltzer P, Chen Y, Jiang Y, Seftor E, Hendrix M, Radmacher M, Simon R, Yakhini Z, Ben-Dor A, Sampas N, Dougherty E, Wang E, Marincola F, Gooden C, Lueders J, Glatfelter A, Pollock P, Carpten J, Gillanders E, Leja D, Dietrich K, Beaudry C, Berens M, Alberts D, Sondak V (2000) Molecular classification of cutaneous malignant melanoma by gene expression profiling. Nature 406: 536–540 10952317 10.1038/35020115

[bib7] Breitling R, Armengaud P, Amtmann A, Herzyk P (2004) Rank products: a simple, yet powerful, new method to detect differentially regulated genes in replicated microarray experiments. FEBS Lett 573: 83–92 15327980 10.1016/j.febslet.2004.07.055

[bib8] Butte A (2002) The use and analysis of microarray data. Nat Rev Drug Discov 1: 951–960 12461517 10.1038/nrd961

[bib9] Cardoso F, Van’t Veer L, Rutgers E, Loi S, Mook S, Piccart-Gebhart MJ (2008) Clinical application of the 70-gene profile: the MINDACT trial. J Clin Oncol 26: 729–735 18258980 10.1200/JCO.2007.14.3222

[bib10] Chang JC, Wooten EC, Tsimelzon A, Hilsenbeck SG, Gutierrez MC, Elledge R, Mohsin S, Osborne CK, Chamness GC, Allred DC, O’Connell P (2003) Gene expression profiling for the prediction of therapeutic response to docetaxel in patients with breast cancer. Lancet 362: 362–369 12907009 10.1016/S0140-6736(03)14023-8

[bib11] Culhane AC, Thioulouse J, Perriere G, Higgins DG (2005) MADE4: an R package for multivariate analysis of gene expression data. Bioinformatics 21: 2789–2790 15797915 10.1093/bioinformatics/bti394

[bib12] Curtin JF, Cotter TG (2004) JNK regulates HIPK3 expression and promotes resistance to Fas-mediated apoptosis in DU 145 prostate carcinoma cells. J Biol Chem 279: 17090–17100 14766760 10.1074/jbc.M307629200

[bib13] de Bruin EC, van de Pas S, Lips EH, van Eijk R, van der Zee MM, Lombaerts M, van Wezel T, Marijnen CA, van Krieken JH, Medema JP, van de Velde CJ, Eilers PH, Peltenburg LT (2005) Macrodissection versus microdissection of rectal carcinoma: minor influence of stroma cells to tumor cell gene expression profiles. BMC Genomics 6: 142 16225673 10.1186/1471-2164-6-142 PMC1283972

[bib14] Delarue FL, Adnane J, Joshi B, Blaskovich MA, Wang DA, Hawker J, Bizouarn F, Ohkanda J, Zhu K, Hamilton AD, Chellappan S, Sebti SM (2007) Farnesyltransferase and geranylgeranyltransferase I inhibitors upregulate RhoB expression by HDAC1 dissociation, HAT association and histone acetylation of the RhoB promoter. Oncogene 26: 633–640 16909123 10.1038/sj.onc.1209819

[bib15] Dennis Jr G, Sherman BT, Hosack DA, Yang J, Gao W, Lane HC, Lempicki RA (2003) DAVID: database for annotation, visualization, and integrated discovery. Genome Biol 4: P3 12734009 PMC193660

[bib16] Eisen MB, Spellman PT, Brown PO, Botstein D (1998) Cluster analysis and display of genome-wide expression patterns. Proc Natl Acad Sci USA 95: 14863–14868 9843981 10.1073/pnas.95.25.14863 PMC24541

[bib17] Emmert-Buck MR, Bonner RF, Smith PD, Chuaqui RF, Zhuang Z, Goldstein SR, Weiss RA, Liotta LA (1996) Laser capture microdissection. Science 274: 998–1001 8875945 10.1126/science.274.5289.998

[bib18] Fellenberg K, Hauser NC, Brors B, Neutzner A, Hoheisel JD, Vingron M (2001) Correspondence analysis applied to microarray data. Proc Natl Acad Sci USA 98: 10781–10786 11535808 10.1073/pnas.181597298 PMC58552

[bib19] Fukino K, Shen L, Patocs A, Mutter GL, Eng C (2007) Genomic instability within tumor stroma and clinicopathological characteristics of sporadic primary invasive breast carcinoma. JAMA 297: 2103–2111 17507346 10.1001/jama.297.19.2103

[bib20] Gentleman RC, Carey VJ, Bates DM, Bolstad B, Dettling M, Dudoit S, Ellis B, Gautier L, Ge Y, Gentry J, Hornik K, Hothorn T, Huber W, Iacus S, Irizarry R, Leisch F, Li C, Maechler M, Rossini AJ, Sawitzki G, Smith C, Smyth G, Tierney L, Yang JY, Zhang J (2004) Bioconductor: open software development for computational biology and bioinformatics. Genome Biol 5: R80 15461798 10.1186/gb-2004-5-10-r80 PMC545600

[bib21] Gershon D (2004) Microarrays go mainstream. Nat Methods 1: 263–270

[bib22] Glas AM, Floore A, Delahaye LJ, Witteveen AT, Pover RC, Bakx N, Lahti-Domenici JS, Bruinsma TJ, Warmoes MO, Bernards R, Wessels LF, Van’t Veer LJ (2006) Converting a breast cancer microarray signature into a high-throughput diagnostic test. BMC Genomics 7: 278 17074082 10.1186/1471-2164-7-278 PMC1636049

[bib23] Guo J, Xiao B, Zhang X, Jin Z, Chen J, Qin L, Mao X, Shen G, Chen H, Liu Z (2004) Combined use of positive and negative immunomagnetic isolation followed by real-time RT-PCR for detection of the circulating tumor cells in patients with colorectal cancers. J Mol Med 82: 768–774 15490093 10.1007/s00109-004-0590-8

[bib24] Hofmann WK, de Vos S, Elashoff D, Gschaidmeier H, Hoelzer D, Koeffler HP, Ottmann OG (2002) Relation between resistance of Philadelphia-chromosome-positive acute lymphoblastic leukaemia to the tyrosine kinase inhibitor STI571 and gene-expression profiles: a gene-expression study. Lancet 359: 481–486 11853794 10.1016/S0140-6736(02)07678-X

[bib25] Huang E, Cheng SH, Dressman H, Pittman J, Tsou MH, Horng CF, Bild A, Iversen ES, Liao M, Chen CM, West M, Nevins JR, Huang AT (2003) Gene expression predictors of breast cancer outcomes. Lancet 361: 1590–1596 12747878 10.1016/S0140-6736(03)13308-9

[bib26] Iizuka N, Oka M, Yamada-Okabe H, Nishida M, Maeda Y, Mori N, Takao T, Tamesa T, Tangoku A, Tabuchi H, Hamada K, Nakayama H, Ishitsuka H, Miyamoto T, Hirabayashi A, Uchimura S, Hamamoto Y (2003) Oligonucleotide microarray for prediction of early intrahepatic recurrence of hepatocellular carcinoma after curative resection. Lancet 361: 923–929 12648972 10.1016/S0140-6736(03)12775-4

[bib27] Jarvis JN, Centola M (2005) Gene-expression profiling: time for clinical application? Lancet 365: 199–200 15652589 10.1016/S0140-6736(05)17754-X

[bib28] Jeffery IB, Higgins DG, Culhane AC (2006) Comparison and evaluation of methods for generating differentially expressed gene lists from microarray data. BMC Bioinformatics 7: 359 16872483 10.1186/1471-2105-7-359 PMC1544358

[bib29] Kwong KY, Bloom GC, Yang I, Boulware D, Coppola D, Haseman J, Chen E, McGrath A, Makusky AJ, Taylor J, Steiner S, Zhou J, Yeatman TJ, Quackenbush J (2005) Synchronous global assessment of gene and protein expression in colorectal cancer progression. Genomics 86: 142–158 15951154 10.1016/j.ygeno.2005.03.012

[bib30] Latza U, Niedobitek G, Schwarting R, Nekarda H, Stein H (1990) Ber-EP4: new monoclonal antibody which distinguishes epithelia from mesothelial. J Clin Pathol 43: 213–219 1692040 10.1136/jcp.43.3.213 PMC502333

[bib31] Li C, Hung Wong W (2001) Model-based analysis of oligonucleotide arrays: model validation, design issues and standard error application. Genome Biol 2: RESEARCH0032 11532216 10.1186/gb-2001-2-8-research0032 PMC55329

[bib32] Liu ET (2007) Stromal effects in breast cancer. N Engl J Med 357: 2537–2538 18094373 10.1056/NEJMp0707576

[bib33] Liu S, Wang H, Currie BM, Molinolo A, Leung HJ, Moayeri M, Basile JR, Alfano RW, Gutkind JS, Frankel AE, Bugge TH, Leppla SH (2008) Matrix metalloproteinase-activated anthrax lethal toxin demonstrates high potency in targeting tumor vasculature. J Biol Chem 283: 529–540 17974567 10.1074/jbc.M707419200 PMC2394502

[bib34] Marton MJ, DeRisi JL, Bennett HA, Iyer VR, Meyer MR, Roberts CJ, Stoughton R, Burchard J, Slade D, Dai H, Bassett Jr DE, Hartwell LH, Brown PO, Friend SH (1998) Drug target validation and identification of secondary drug target effects using DNA microarrays. Nat Med 4: 1293–1301 9809554 10.1038/3282

[bib35] Michel C, Desdouets C, Sacre-Salem B, Gautier JC, Roberts R, Boitier E (2003) Liver gene expression profiles of rats treated with clofibric acid: comparison of whole liver and laser capture microdissected liver. Am J Pathol 163: 2191–2199 14633594 10.1016/S0002-9440(10)63577-8 PMC1892366

[bib36] Moyers JS, Bilan PJ, Zhu J, Kahn CR (1997) Rad and Rad-related GTPases interact with calmodulin and calmodulin-dependent protein kinase II. J Biol Chem 272: 11832–11839 9115241 10.1074/jbc.272.18.11832

[bib37] Nyengaard JR (1999) Stereologic methods and their application in kidney research. J Am Soc Nephrol 10: 1100–1123 10232698 10.1681/ASN.V1051100

[bib38] Patocs A, Zhang L, Xu Y, Weber F, Caldes T, Mutter GL, Platzer P, Eng C (2007) Breast-cancer stromal cells with TP53 mutations and nodal metastases. N Engl J Med 357: 2543–2551 18094375 10.1056/NEJMoa071825

[bib39] Ramaswamy S, Ross KN, Lander ES, Golub TR (2003) A molecular signature of metastasis in primary solid tumors. Nat Genet 33: 49–54 12469122 10.1038/ng1060

[bib40] Ramaswamy S, Tamayo P, Rifkin R, Mukherjee S, Yeang CH, Angelo M, Ladd C, Reich M, Latulippe E, Mesirov JP, Poggio T, Gerald W, Loda M, Lander ES, Golub TR (2001) Multiclass cancer diagnosis using tumor gene expression signatures. Proc Natl Acad Sci USA 98: 15149–15154 11742071 10.1073/pnas.211566398 PMC64998

[bib41] Ross DT, Scherf U, Eisen MB, Perou CM, Rees C, Spellman P, Iyer V, Jeffrey SS, Van de Rijn M, Waltham M, Pergamenschikov A, Lee JC, Lashkari D, Shalon D, Myers TG, Weinstein JN, Botstein D, Brown PO (2000) Systematic variation in gene expression patterns in human cancer cell lines. Nat Genet 24: 227–235 10700174 10.1038/73432

[bib42] Schroeder A, Mueller O, Stocker S, Salowsky R, Leiber M, Gassmann M, Lightfoot S, Menzel W, Granzow M, Ragg T (2006) The RIN: an RNA integrity number for assigning integrity values to RNA measurements. BMC Mol Biol 7: 3 16448564 10.1186/1471-2199-7-3 PMC1413964

[bib43] Seibenhener ML, Geetha T, Wooten MW (2007) Sequestosome 1/p62 – more than just a scaffold. FEBS Lett 581: 175–179 17188686 10.1016/j.febslet.2006.12.027 PMC1850379

[bib44] Selaru FM, Xu Y, Yin J, Zou T, Liu TC, Mori Y, Abraham JM, Sato F, Wang S, Twigg C, Olaru A, Shustova V, Leytin A, Hytiroglou P, Shibata D, Harpaz N, Meltzer SJ (2002) Artificial neural networks distinguish among subtypes of neoplastic colorectal lesions. Gastroenterology 122: 606–613 11874992 10.1053/gast.2002.31904

[bib45] Shipp MA, Ross KN, Tamayo P, Weng AP, Kutok JL, Aguiar RC, Gaasenbeek M, Angelo M, Reich M, Pinkus GS, Ray TS, Koval MA, Last KW, Norton A, Lister TA, Mesirov J, Neuberg DS, Lander ES, Aster JC, Golub TR (2002) Diffuse large B-cell lymphoma outcome prediction by gene-expression profiling and supervised machine learning. Nat Med 8: 68–74 11786909 10.1038/nm0102-68

[bib46] Smith MJ, Coffey JC, Wang JH, Cotter TG, Redmond HP (2003) Gene expression profiling in biliary atresia. Lancet 361: 971–972; 10.1016/s0140-6736(03)12757-2 12648998

[bib47] Sugiyama Y, Sugiyama K, Hirai Y, Akiyama F, Hasumi K (2002) Microdissection is essential for gene expression profiling of clinically resected cancer tissues. Am J Clin Pathol 117: 109–116 11789716 10.1309/G1C8-39MF-99UF-GT2K

[bib48] Suzuki A, Nakauchi H, Taniguchi H (2004) Prospective isolation of multipotent pancreatic progenitors using flow-cytometric cell sorting. Diabetes 53: 2143–2152 15277399 10.2337/diabetes.53.8.2143

[bib49] van ‘t Veer LJ, Dai H, van de Vijver MJ, He YD, Hart AA, Mao M, Peterse HL, van der Kooy K, Marton MJ, Witteveen AT, Schreiber GJ, Kerkhoven RM, Roberts C, Linsley PS, Bernards R, Friend SH (2002) Gene expression profiling predicts clinical outcome of breast cancer. Nature 415: 530–536 11823860 10.1038/415530a

[bib50] van de Vijver MJ, He YD, van’t Veer LJ, Dai H, Hart AA, Voskuil DW, Schreiber GJ, Peterse JL, Roberts C, Marton MJ, Parrish M, Atsma D, Witteveen A, Glas A, Delahaye L, van der Velde T, Bartelink H, Rodenhuis S, Rutgers ET, Friend SH, Bernards R (2002) A gene-expression signature as a predictor of survival in breast cancer. N Engl J Med 347: 1999–2009 12490681 10.1056/NEJMoa021967

[bib51] Vider BZ, Zimber A, Hirsch D, Estlein D, Chastre E, Prevot S, Gespach C, Yaniv A, Gazit A (1997) Human colorectal carcinogenesis is associated with deregulation of homeobox gene expression. Biochem Biophys Res Commun 232: 742–748 9126347 10.1006/bbrc.1997.6364

[bib52] Waguri N, Suda T, Nomoto M, Kawai H, Mita Y, Kuroiwa T, Igarashi M, Kobayashi M, Fukuhara Y, Aoyagi Y (2003) Sensitive and specific detection of circulating cancer cells in patients with hepatocellular carcinoma; detection of human telomerase reverse transcriptase messenger RNA after immunomagnetic separation. Clin Cancer Res 9: 3004–3011 12912949

[bib53] Wang Y, Jatkoe T, Zhang Y, Mutch MG, Talantov D, Jiang J, McLeod HL, Atkins D (2004) Gene expression profiles and molecular markers to predict recurrence of Dukes’ B colon cancer. J Clin Oncol 22: 1564–1571 15051756 10.1200/JCO.2004.08.186

[bib54] Winegarden N (2003) Microarrays in cancer: moving from hype to clinical reality. Lancet 362: 1428 14602430 10.1016/S0140-6736(03)14724-1

[bib55] Zigeuner RE, Riesenberg R, Pohla H, Hofstetter A, Oberneder R (2000) Immunomagnetic cell enrichment detects more disseminated cancer cells than immunocytochemistry *in vitro*. J Urol 164: 1834–1837 11025779

